# Barriers to Achieving the Recommended Duration of Breastfeeding in Women Visiting the Well-Baby Clinic of King Abdulaziz University Hospital: A Cross-Sectional Study

**DOI:** 10.7759/cureus.61257

**Published:** 2024-05-28

**Authors:** Eman Alkhalawi, Waad S Alhumaidi, Reema W Alharbi, Renad M Alsuhaimi, Shroug A Alsayed, Heidi Al-Wassia

**Affiliations:** 1 Department of Family and Community Medicine, Faculty of Medicine in Rabigh, King Abdulaziz University, Rabigh, SAU; 2 Medical College, Faculty of Medicine, King Abdulaziz University, Jeddah, SAU; 3 Department of Pediatrics, King Abdulaziz University Hospital, Jeddah, SAU

**Keywords:** women, mothers, barriers, duration of breastfeeding, breastfeeding

## Abstract

Background

‏Natural breast milk is the ideal food for infants. Exclusive breastfeeding for the first six months and continuation of breastfeeding for the first year is recommended. However, less than half of infants worldwide are breastfed for six months.

Objectives

We sought to explore the discrepancy between the recommended and achieved duration of breastfeeding in a sample of mothers in Jeddah, Saudi Arabia, and the barriers to achieving the recommended duration of breastfeeding. We also examine the association between demographic and birth-related variables and breastfeeding initiation and duration.

Methods

This was a cross-sectional study that took place at the well-baby clinic of King Abdulaziz University Hospital (KAUH). Face-to-face interviews were conducted with 38 women who were visiting for the routine vaccination of their infants. The association between demographic and birth-related variables and breastfeeding was explored using the chi-square test.

Results

A total of 31 (81.6%) of the mothers breastfed their babies. Of those, only 44% (n = 11) breastfed for six months or longer. Among the mothers who were still breastfeeding, they planned to breastfeed for one year on average (12.2 ± 5.0 months). Among the mothers who were not breastfeeding at the time of the study, the mean duration of breastfeeding was only 3.7 months (SD = 4.6 months). A total of 92% of mothers introduced breast milk alternatives, and on average, it was introduced during the second month (1.8 ± 3.3 months). The main obstacles that led the mothers to stop breastfeeding were the child’s illness (87.5%), decreased milk production (41.7%), and the child refusing to breastfeed (25.0%). Younger maternal age and initiation of breastfeeding within 24 hours of birth were positively associated with breastfeeding, while the introduction of breast milk alternatives from birth was negatively associated with breastfeeding. Only younger maternal age was significantly associated with breastfeeding for longer than six months.

Conclusions

Although many mothers breastfed their children initially, the duration of breastfeeding was short. Teaching and encouraging mothers about the benefits of breastfeeding and proper nursing techniques and addressing common barriers may help increase the duration of breastfeeding.

## Introduction

Breastfeeding is the feeding of infants directly from female human breast milk. ‏Breast milk is the ideal food for infants. It provides them with all the nutrients for healthy growth. As children grow, breast milk adapts to meet their changing needs, provides passive immunity from many infectious diseases, and is accessible and available whenever the need arises. Breastfeeding is one of the key factors in creating a strong relationship between the mother and the child. It is a significant modifiable factor affecting health outcomes for both mothers and their infants. One of the many benefits is that it decreases infectious disease mortality among infants and children who are exclusively breastfed. Children who are not breastfed are more susceptible to a range of diseases, including obesity, type 1 and type 2 diabetes, leukemia, and sudden infant death syndrome. On the maternal side, it improves family planning and reduces the risk of type 2 diabetes mellitus, metabolic syndrome, ovarian cancer, and breast cancer [1]. Yet, it is dose-dependent, meaning with exclusivity and longer duration, these benefits will increase.

Breast milk is widely acknowledged as the ideal source of nutrition for human infants, with exclusive breastfeeding for the initial six months of life being advocated by numerous national and international organizations such as the World Health Organization (WHO) and the United Nations International Children's Emergency Fund (UNICEF) [2]. The WHO specifically advises initiating breastfeeding within the first hour after birth and continuing it for the first six months, with continuation up to two years or beyond. The Saudi Ministry of Health also advocates for these recommendations [3]. However, despite extensive global campaigns to promote breastfeeding, only 44% of infants under six months are breastfed worldwide, as reported by the UNICEF in 2019 [4].

A study covering five geographic regions in Saudi Arabia showed that 8.3% of mothers never breastfeed. Breastfeeding was initiated within an hour of birth for 43.6% of women: 26% in the northern region, 38.4% in the central region, 45% in the western region, 49% in the eastern region, and 63% in the southern region [5].

The journey of breastfeeding is an easy journey for some and a difficult one for others. We explore the discrepancy between the achieved duration of breastfeeding and that recommended in a sample of mothers in Jeddah, Saudi Arabia, and the barriers to achieving the recommended duration of breastfeeding. We also examine the association between both breastfeeding and breastfeeding duration with demographic and birth history variables.

## Materials and methods

We conducted a cross-sectional face-to-face interview at the well-baby clinic of King Abdulaziz University Hospital (KAUH) with women who were visiting for the routine vaccination of their infants. We included women who were older than 18 years and who gave written consent and excluded women who had undergone bilateral mastectomy or breast reduction surgery.

Raosoft software (Raosoft, Inc., Seattle, WA) was used to calculate the sample size with a 5% margin of error and 95% confidence level, based on a population size of 200 (an estimate of the number of women visiting the well-baby clinic at KAUH over one month). The response of the distribution was set as 97% based on previous literature on the number of women who initiate breastfeeding. A sample size of 37 mothers was required. Ethical approval was obtained from King Abdulaziz University Hospital’s ethical committee (No.: 468-22).

The questionnaire was divided into three sections. The first section included patient demographic information (e.g., age, education level, income level, and employment). Income was inquired about subjectively by asking participants to select their income level (low, intermediate, and high), due to cultural reluctance to share income or lack of knowledge of women about their husband’s income. The second section focused on the birth and health history. The third section asked about breastfeeding history.

We interviewed 38 participants face-to-face. The data were entered into a Google Forms (Google, Mountain View, CA) survey template and were then extracted into a spreadsheet and imported into Stata SE version 18 (StataCorp LLC, College Station, TX) for analysis. We described the continuous data (age, duration of breastfeeding) as means and standard deviation, and categorical data by frequency and percentage. We then explored the factors associated with failing to establish breastfeeding and to achieve the recommended duration of breastfeeding (demographic variables and medical and birth history) using the chi-square test at a 0.05 significance level.

## Results

A total of 38 women participated in the study. The mean age of the mothers participating in the study was 33.1 (SD = 7.01) years, and the mean age of their children was 15.9 months (SD = 18.1), ranging between one and 58 months (Table 1). About half of the mothers had a university degree, followed by a high school diploma. Most (N = 29, 76%) of the mothers were not employed, with the majority being housewives and two (5.2%) being students. Among those who had a job, the largest proportion (N = 3, 27.3%) had a maternity leave of 70 days, but some had as little as 30 days. Most of the participants (N = 31, 81.6%) were in the intermediate income group. Additionally, 36 (94.7%) of the participants were non-smokers. For 28 (73.7%) of the participants, the child they had during the time of the study was not their first child, with 24 (64.2%) having one to three children, including the current child. The majority (N = 33, 86.8%) had given birth to their child without experiencing complications. None of the participants had undergone mastectomy.

**Table 1:** Demographic characteristics of the participants (N = 38). The data have been represented as N and % for categorical data and mean ± SD for continuous data.

Variable	N	%
Maternal age, years (mean ± SD)	33.08 ± 7.01	
Infant age, months (mean ± SD)	15.9 ± 18.1	
Education level		
Elementary school	3	7.9
Middle school	2	5.3
High school	13	34.2
University	20	52.6
Employed		
Yes	9	23.7
No	29	76.3
Job		
Business management	2	5.3
Doctor	2	5.3
Teacher	3	7.9
Housewife	27	71.1
Nurse	1	2.6
Psychologist	1	2.6
Student	2	5.3
Duration of maternity leave		
30 days	2	18.2
60 days	1	9.1
70 days	3	27.3
90 days	2	18.2
Not applicable	2	18.2
Income level		
Low income	4	10.5
Intermediate income	31	81.6
High income	3	7.9
Smoking		
Yes	2	5.3
No	36	94.7
First child		
Yes	10	26.3
No	28	73.7
Number of births (with the current child)		
1-3 children	24	64.2
4-6 children	11	28.9
7-9 children	3	7.9
Complications during the birth of this child		
Yes	5	13.2
No	33	86.8
Type of complications		
Bleeding	2	5.3
Fever	2	5.3
Seizures	1	2.6
Meconium aspiration	1	2.6
Vaginal infection	1	2.6
None	33	86.8
Mastectomy		
Yes	0	0
No	38	100

Most (N = 31, 81.6%) of the mothers breastfed their babies and 65.8% (N = 25) of the mothers started breastfeeding within 24 hours after giving birth (Table 2). Of the participants, 31.6% (N = 12) were still breastfeeding during the time of the study (including only 11 of the 20 children that were aged 12 months or younger (45%)). Among the mothers who were still breastfeeding, they planned to breastfeed for one year on average (12.2 ± 5.0 months). On the other hand, among the mothers who were not breastfeeding at the time of the study, they breastfed for only 3.7 months on average (SD = 4.56). In addition, almost all participants had used breast milk alternatives, and these were introduced mostly at birth (N = 14, 37%), and before two months on average.

**Table 2:** Feeding history among the participants (N = 38). The data have been represented as N and % for categorical data and mean ± SD for continuous data.

Variables	N	%
Did you start trying to breastfeed within 24 hours after giving birth?		
Yes	25	65.8
No	13	34.2
Did you breastfeed?		
Yes	31	81.6
No	7	18.1
Are you currently breastfeeding?		
Yes	12	31.6
No	26	68.4
Until what age do you plan to breastfeed (months)? (N = 12) (mean ± SD)	12.2	5.0
How long was the breastfeeding period (months)? (N = 18) (mean ± SD)	3.7	4.6
Did you give your infant any breast milk alternative (formula)?		
Yes	34	91.9
No	3	8.1
Age of introducing breast milk alternative (months). (N = 34) (mean ± SD)	1.8	3.3
Did you introduce solid food?		
Yes	28	73.6
No	10	26.3

**Figure 1:**
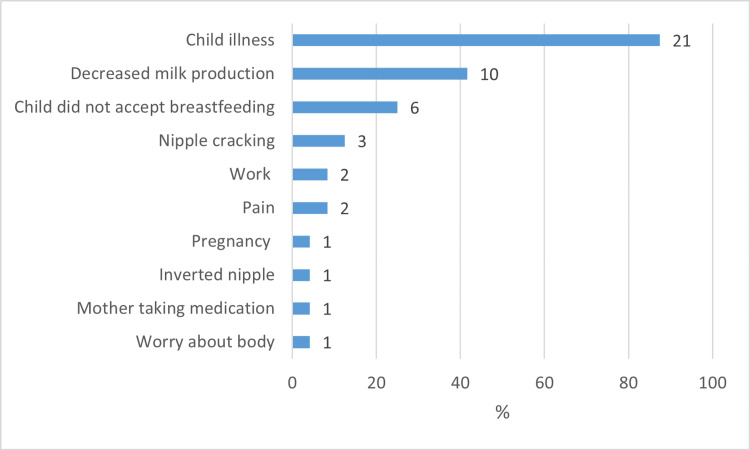
Reasons mothers stopped breastfeeding before 12 months (n = 24).

Among mothers whose child was not breastfed or breastfed for less than a year (N = 24), the main obstacles that led the mothers to stop breastfeeding before 12 months were the child’s illness (N = 21, 87.5%), decreased milk production (N = 10, 41.7%), and the child refusing to breastfeed (N = 6, 25.0%) (Figure 1). Ten mothers gave more than one reason.

**Figure 2:**
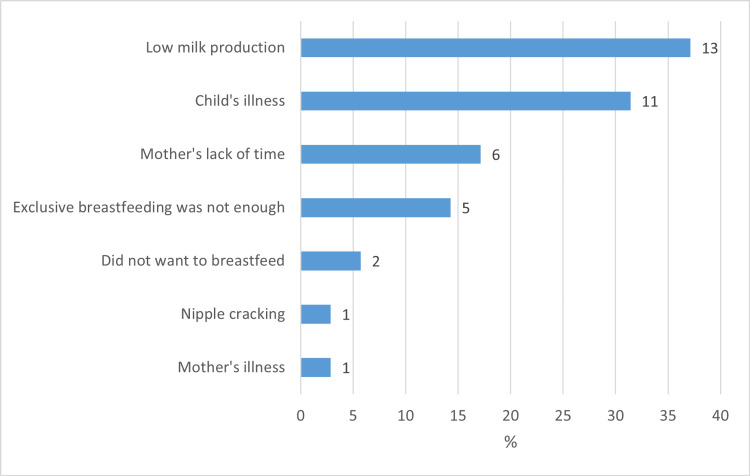
Reasons for introducing breast milk alternatives (n = 35).

The main reasons for introducing breast milk alternatives were decreased milk production (N = 13, 37%), child’s illness (N = 11, 31.40%), mother’s lack of time (N = 6, 17.1%), and the perception that exclusive breastfeeding was not enough for the infant (N = 5, 14.3%) (Figure 2). Eight mothers gave more than one reason for introducing breast milk alternatives.

There was a statistically significant relationship between the mother's age and breastfeeding (Table 3). While 93% (N = 13) of mothers under 30 years breastfed, none of the mothers over 40 years breastfed (p < 0.001). Mothers who initiated breastfeeding within 24 hours of birth were also more likely to breastfeed (p = 0.001), while those who introduced formula from birth were less likely to breastfeed (p = 0.012). These differences were not statistically significant when looking at breastfeeding for at least six months as an outcome (Table 4).

**Table 3:** Association between demographic and feeding history variables and breastfeeding (n = 38). The data are presented as N and %. P-values of the chi-square test are considered significant at the 5% level.

	Breastfed				
	Yes (N = 31)		No (N = 7)		P-value of the chi-square test
	N	%	N	%	
Maternal age					
Younger than 30	13	92.9	1	7.1	
30-34	9	90	1	10	
35-40	9	100	0	0	
40+	0	0	4	100	<0.001
Education					
Elementary school	2	66.7	1	33.3	
Middle school	0	0	1	100	
High school	10	76.9	3	23.1	
University	17	89.5	2	10.5	0.134
Employment					
Yes	9	90	1	10	
No	22	78.6	6	21.43	0.424
Income					
Low	4	100	0	0	
Medium	25	80.6	6	19.4	
High	2	66.7	1	33.3	0.505
Smoking					
Yes	2	100	0	0	
No	29	80.6	7	19.4	0.49
First child					
Yes	10	100	0	0	
No	21	75	7	25	0.08
Birth complications					
Yes	5	100	0	0	
No	26	78.8	7	21.2	0.254
Initiated breastfeeding within 24 hours					
Yes	24	96	1	4	
No	7	53.8	6	46.2	0.001
Introduced breast milk alternative from birth					
Yes	7	58.3	5	41.7	
No	24	81.6	2	7.7	0.012

**Table 4:** Association between demographic and feeding history variables and breastfeeding for more versus less than six months (n = 25). The data are presented as N and %.P-values of the chi-square test are considered significant at the 5% level.

	More than six months (N = 11)		Less than six months (N = 14)		P-value of the chi-square test
	N	%	N	%	
Maternal age (years)					
Younger than 30	2	18.2	9	81.8	
30-34	4	57.1	3	42.9	
35-40	5	71.4	2	28.6	
40+	0	100	0	0	0.061
Education					
Elementary school	1	50	1	50	
Middle school	0	0	0	0	
High school	1	14.3	6	85.7	
University	9	64.3	6	35.7	0.133
Employment					
Yes	2	33.3	4	66.7	
No	9	47.4	10	52.6	0.546
Income				0	
Low	0	0	2	100	
Medium	10	45.5	12	54.6	
High	1	100	0	0	0.239
Smoking					
Yes	0	0	2	100	
No	11	44	12	56	0.191
First child		0			
Yes	3	42.9	4	57.1	
No	8	47.1	9	52.9	0.851
Birth complications					
Yes	1	33.3	2	66.7	
No	10	45.5	12	54.6	0.692
Initiated breastfeeding within 24 hours					
Yes	8	42.1	11	57.9	
No	3	50	3	50	0.734
Introduced breast milk alternatives from birth					
Yes	1	16.7	5	83.3	
No	10	52.6	9	47.4	0.122

## Discussion

The study aimed to identify barriers to achieving the recommended duration of breastfeeding for a sample of women in Jeddah. The study findings reveal that 81.6% (N = 31) of the mothers breastfed their babies. However, during the time of the study, only 45% of infants one year or younger were still breastfed. Among the mothers who were still breastfeeding, most of them planned to breastfeed for a total of one year from birth on average. Mothers who were not breastfeeding at the time of the study breastfed on average for approximately four months only.

Further, we found that the main obstacles that led the mothers to stop breastfeeding were the child’s illness, followed by decreased milk production and the child refusing to breastfeed.

Similar to these findings, a study in the Mecca region found that 81% of mothers breastfed their infants. Failure to establish early breastfeeding was associated with using pacifiers, mothers not being informed of the importance of breastfeeding, the introduction of formula early on, and the practice of not rooming infants in the mother's room [6]. In contrast, a study from the UK found that the main reasons for discontinuation of breastfeeding were the mother's attitudes toward feeding her infant, negative breastfeeding challenges during the first four weeks of the baby's life, smoking by the mother, using a pacifier, and returning to work early [7].

Most mothers introduced breast milk alternatives (formula) for their babies, many of whom introduced it from the time of birth or soon thereafter during the first two months. The most common reason given by mothers was low milk production. A prospective study from Jeddah, Saudi Arabia reported that only 37.5% of infants were exclusively breastfed for the first two months [8]. A study from India reported that skin-to-skin contact and breastfeeding self-efficacy were important determinants of perceived milk supply, which in turn was positively associated with exclusive breastfeeding [9]. Therefore, these factors should be explored in future studies in this population.

While some of the reasons given for stopping breastfeeding may have posed real impediments, they may also reflect misconceptions held by mothers. Very few childhood illnesses necessitate the interruption of breastfeeding, and continued breastfeeding is beneficial for many common childhood infections as it provides passive immunity and prevents dehydration. Unrealistic expectations of breastfeeding frequency and duration or infant weight gain may lead women to believe their milk production is insufficient, causing a cycle of formula supplementation and decreased milk production or refusal of the child to latch to the breast. Learning correct nursing techniques is vital to avoid pain and distress that lead to mothers giving up breastfeeding. In addition, maternal motivation may play a role in continuing breastfeeding. A randomized controlled trial that offered a brief motivational intervention to women in the immediate postpartum period showed increased breastfeeding duration in the intervention group compared to controls who only received education [10].

Our study found a statistically significant relationship between the mother's age and breastfeeding. Mothers under 30 years were more likely to breastfeed and breastfeed their child longer than mothers who were aged over 40 years. Similarly, a study conducted in Jeddah found that early cessation of breastfeeding is associated with maternal age and health status, mothers' knowledge and attitude toward breastfeeding, and modifiable challenges encountered in the hospital such as latching problems [8]. A study in Zimbabwe noted that young mothers get more support from their spouses during the breastfeeding period, which makes them breastfeed for the period recommended by the health professional as opposed to old mothers who have given birth more than once [11]. Higher awareness of the benefits of breastfeeding and changing social norms among younger cohorts of women in response to more recent breastfeeding promotion campaigns may have also played a role in the observed association.

This finding is in line with the findings in a study in Ethiopia, which established factors associated with starting breastfeeding within an hour of birth, including the age of the mother and the mother's education. The study noted that younger mothers start breastfeeding immediately after delivery due to anxiety about parenthood [12].

Various obstacles to breastfeeding that were not explored in this study were identified in the literature. For example, a study in Iran identified a lack of social support, mothers' self-efficacy, preparedness of staff, and breastfeeding privacy as barriers [13]. Therefore, it is worth noting that barriers to mothers’ breastfeeding vary depending on the situation in which they find themselves during the period of breastfeeding.

The cross-sectional design may have affected women’s accurate recollection of the duration of breastfeeding history. To mitigate the recall bias, the age range of the children involved was limited to less than six years. The sample size was calculated based on the primary outcome, which was the initiation of breastfeeding, and the volume of the well-baby clinic, which is modest. The study therefore may have been underpowered to detect some associations between the measured variables and outcomes, especially the duration of breastfeeding. Due to the setting of the university hospital, generalizations cannot be made for the Saudi population as a whole. However, the alarmingly short average duration of breastfeeding despite a moderately high rate of initiation, along with a high proportion of women who introduced breast milk alternatives soon after birth warrant further exploration in various settings and in more detail.

## Conclusions

Although many mothers breastfed their children at first, the duration of breastfeeding on average was short. The child's illness, decreased milk production, work commitments, the child's refusal to be breastfed, and the mother's fatigue and stress all served as barriers that kept mothers from continuing to breastfeed. The role of the common practice of early introduction of breast milk alternatives in impeding the success of breastfeeding should also be further explored.

Teaching women about the benefits of breastfeeding and proper nursing techniques, and providing personal and logistical support to address barriers to exclusive breastfeeding on a policy and institutional level could help increase the duration of breastfeeding among women in Saudi Arabia.
